# Translation of COVID-19 Serology Test on Foil-Based Lateral Flow Chips: A Journey from Injection Molding to Scalable Roll-to-Roll Nanoimprint Lithography

**DOI:** 10.3390/bios15040229

**Published:** 2025-04-04

**Authors:** Pakapreud Khumwan, Stephan Ruttloff, Johannes Götz, Dieter Nees, Conor O’Sullivan, Alvaro Conde, Mirko Lohse, Christian Wolf, Nastasia Okulova, Janine Brommert, Richard Benauer, Ingo Katzmayr, Nikolaus Ladenhauf, Wilfried Weigel, Maciej Skolimowski, Max Sonnleitner, Martin Smolka, Anja Haase, Barbara Stadlober, Jan Hesse

**Affiliations:** 1 JOANNEUM RESEARCH Materials, Institute for Sensors, Photonics and Manufacturing Technologies, Franz-Pichler-Strasse 30, 8160 Weiz, Austria; pakapreud.khu@biotec.or.th (P.K.); stephan.ruttloff@joanneum.at (S.R.); johannes.goetz@joanneum.at (J.G.); dieter.nees@joanneum.at (D.N.); christian.wolf@joanneum.at (C.W.); martin.smolka@joanneum.at (M.S.); anja.haase@joanneum.at (A.H.); barbara.stadlober@joanneum.at (B.S.); 2 Inmold A/S, Teglbuen 10, 2990 Nivå, Denmark; co@inmold.dk (C.O.); no@inmold.dk (N.O.); 3 Micronit B.V., Colosseum 15, 7521 PV Enschede, The Netherlands; alvaro.conde@micronit.com (A.C.); maciej.skolimowski@micronit.com (M.S.); 4 Micro Resist Technology GmbH, Koepenicker Strasse 325, 12555 Berlin, Germany; m.lohse@microresist.de; 5 Temicon GmbH, Konrad-Adenauer-Allee 11, 44263 Dortmund, Germany; brommert@temicon.de; 6 Bionic Surface Technologies GmbH, Liebenauer Hauptstrasse 2-6, 8041 Graz, Austria; benauer@bionicsurface.com; 7 GENSPEED Biotech GmbH, Gewerbepark 2, 4261 Rainbach im Mühlkreis, Austria; ingo.katzmayr@genspeed-biotech.com (I.K.); nikolaus.ladenhauf@medunigraz.at (N.L.); max.sonnleitner@genspeed-biotech.com (M.S.); 8 Scienion GmbH, Wagner-Régeny-Strasse 15, 12489 Berlin, Germany; w.weigel@scienion.com; 9 Microfluidics Innovation Hub, Franz-Pichler-Strasse 30, 8160 Weiz, Austria

**Keywords:** lab-on-a-chip, point-of-care diagnostics, roll-to-roll manufacturing, lateral flow test, UV nanoimprint lithography, extrusion coating

## Abstract

Lateral flow tests (LFTs) had a pivotal role in combating the spread of the SARS-CoV-2 virus throughout the COVID-19 pandemic thanks to their affordability and ease of use. Most of LFT devices were based on nitrocellulose membrane strips whose industrial upscaling to billions of devices has already been extensively demonstrated. Nevertheless, the assay option in an LFT format is largely restricted to qualitative detection of the target antigens. In this research, we surveyed the potential of UV nanoimprint lithography (UV-NIL) and extrusion coating (EC) for the high-throughput production of disposable capillary-driven, foil-based tests that allow multistep assays to be implemented for quantitative readout to address the inherent lack of on-demand fluid control and sensitivity of paper-based devices. Both manufacturing technologies operate on the principle of imprinting that enables high-volume, continuous structuring of microfluidic patterns in a roll-to-roll (R2R) production scheme. To demonstrate the feasibility of R2R-fabricated foil chips in a point-of-care biosensing application, we adapted a commercial chemiluminescence multiplex test for COVID-19 antibody detection originally developed for a capillary-driven microfluidic chip manufactured with injection molding (IM). In an effort to build a complete ecosystem for the R2R manufacturing of foil chips, we also recruited additional processes to streamline chip production: R2R biofunctionalization and R2R lamination. Compared to conventional fabrication techniques for microfluidic devices, the R2R techniques highlighted in this work offer unparalleled advantages concerning improved scalability, dexterity of seamless handling, and significant cost reduction. Our preliminary evaluation indicated that the foil chips exhibited comparable performance characteristics to the original IM-fabricated devices. This early success in assay translation highlights the promise of implementing biochemical assays on R2R-manufactured foil chips. Most importantly, it underscores the potential utilization of UV-NIL and EC as an alternative to conventional technologies for the future development in vitro diagnostics (IVD) in response to emerging point-of-care testing demands.

## 1. Introduction

**Figure 1:**
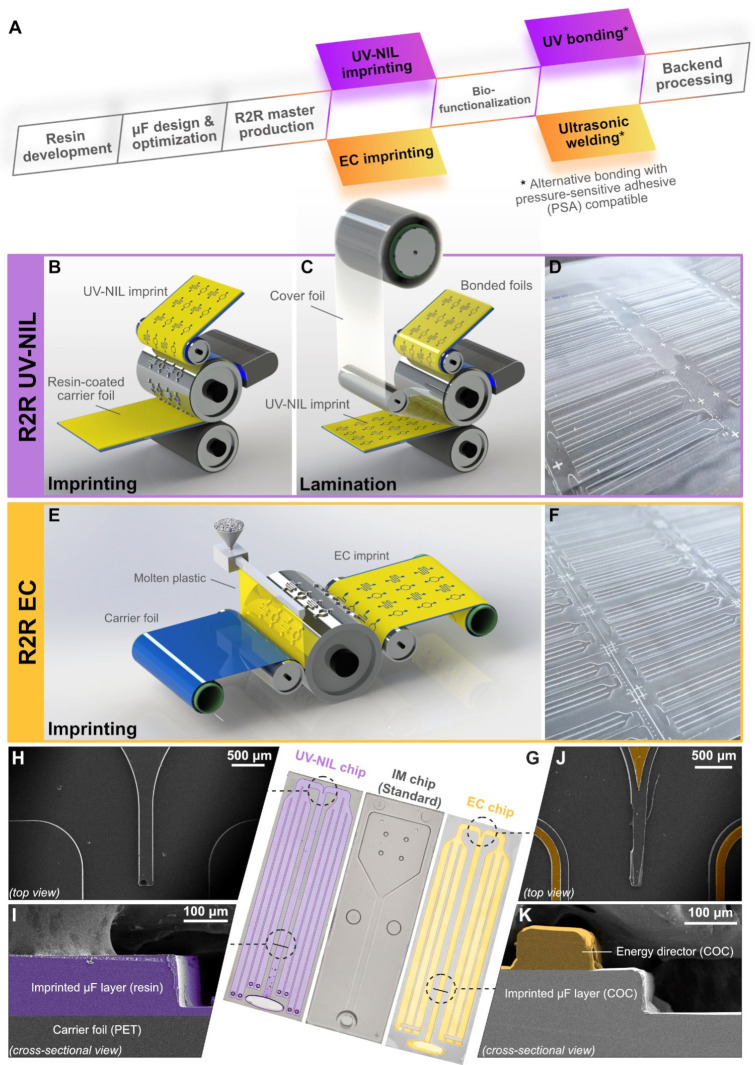
(**A**) A schematic of the high-throughput fabrication process of foil chips for lateral flow assay applications. In this work, we exploited UV nanoimprint lithography (UV-NIL) and extrusion coating (EC) in parallel to develop diagnostic chips for implementing a model µ-ELISA test for COVID-19 antibody. (**B**,**C**) Illustrations of the UV-based imprinting and lamination methods that utilize photocuring of UV curable resins. (**D**) An example of UV-NIL microfluidic chips being imprinted on a roll of carrier PET foil. (**E**) An illustration of the extrusion coating imprinting that utilizes molten thermoplastic to directly form microfluidic structures, which will be hardened upon cooling. (**F**) An example of EC microfluidic chips based on cyclic olefin copolymer (COC). (**G**) Side-by-side comparison of foil chips with the standard GENSPEED chip manufactured with injection molding (IM). The sensing channel is located in the middle in all variants of chips. (**H**,**I**) Scanning electron micrographs of UV-NIL chips highlighting the T-junction where the waste channels branch off from the sensing channel (center), and a cross section of the imprint whose depth is ca. 125 µm (purple). (**J**,**K**) Scanning electron micrographs of EC chips highlighting the T-junction where the waste channels branch off from the sensing channel (center), and a cross section of the imprint whose depth is ca. 200 µm. The microfluidic features of EC chips include an additional layer of 100-µm tall energy director (yellow) to facilitate ultrasonic welding for chip lamination.

A high demand for testing during the active onset of the COVID-19 pandemic had catapulted the development of in vitro diagnostics (IVD) to address the need for population-scale testing in both centralized and decentralized settings [1]. To facilitate mass testing, several paper-based lateral flow tests (LFTs) assumed a pivotal role in curbing the spread of the SARS-CoV-2 virus thanks to their key merits of being affordable and easy to operate [2,3]. These attributes made disposable LFTs highly accessible to the general population, as reflected by a total of 3 billion estimated tests worldwide, a significant portion of which is expected to be shared by LFTs [4]. It goes without saying that an unprecedented demand for LFT use inevitably called for scalable production. Herein, we present parallel developments of foil-based capillary driven microfluidic LFT chip production by exploiting two high-throughput roll-to-roll (R2R) manufacturing technologies. To achieve this goal, we harnessed UV nanoimprint lithography (UV-NIL) and extrusion coating (EC) as viable alternatives to conventional injection molding to create two model pilot fabrication schemes (Figure 1A) that enable the streamlined, high-volume large-area microstructuring [5] of foil-based microfluidic chips (throughput higher than 200 chips/min) for conducting multiplex COVID-19 antibody test in the lateral flow format.

The onset of the recent COVID-19 pandemic has heralded new advances in testing technologies that are tailored for different purposes, target users, and scales of testing with a common goal of identifying the presence of SARS-CoV-2 virus. Given the dynamic at which the pandemic unfolded itself across multiple waves afflicting scores of individuals, we have seen a host of LFT-formatted immunoassays, often paper-based, that emerged as simplified point-of-care screening tools. These tests complement centralized reverse transcription quantitative polymerase chain reaction (RT-qPCR) tests by improving the aspects of cost-effectiveness, turnaround time, and testing equity. Nearly all LFTs in the global market were manufactured based on low-cost nitrocellulose paper blotted with probe molecules, creating a two-dimensional flow vessel to interrogate the presence of specific analytes in the test samples. While the majority of commercially available rapid LFTs are antigen tests designed to qualitatively detect SARS-CoV-2 proteins [3,6], a small fraction of the product landscape is shared by serologic (antibody) tests that also evolved in the same format. In contrast to the antigen test, the antibody test interrogates the profile of immune responses to the past COVID-19 infection and vaccinations through the detection of specific serum antibodies [7,8] via a miniaturized analog of the well-established sandwich enzyme-linked immunosorbent assay (µ-ELISA) [9].

Despite their apparent benefits, rapid paper-based LFTs have technical shortcomings, e.g., a lack of sensitivity, that restrict their utilization in quantitative analysis. Recent studies conducted within the scope of the COVID-19 test alone have highlighted that paper-based devices can vary significantly in their performance across assay developers [10]. One fundamental limitation of paper that partially undertones such variations between test kits is attributed to the intrinsic heterogeneity of porous nitrocellulose that is difficult to standardize [11]. This poses a direct problem to the surface biofunctionalization and flow control [12,13]. Paper-based devices also have a limited fluid capacity of under 100 µL [14,15,16], which is inadequate for accommodating multistep quantitative analysis. Although several quantitative optical [17,18,19,20,21] and electrochemical [22,23,24,25,26] readout strategies to improve the sensitivity have been reported, this inherent heterogeneity places a limit on its ability to generate reliable quantitative signals to be measured on paper-based LFT devices. Since antibody testing inherently demands a mechanism for quantitative result reporting, it is challenging to implement this type of μ-ELISA assay on paper-based systems unless a highly elaborated fluid control system is integrated [27].

To circumvent this issue, plastic substrates with microstructures for liquid transport have been utilized as an alternative immobilization substrate and flow carrier to paper in the fabrication of foil-based microfluidics. The compatibility of these devices for biochemical assays has been demonstrated through chemiluminescence [28], colorimetric [29], and electrochemical [30] detections of bioanalytes. In terms of manufacturing, synthetic polymers offer a better control of material’s chemical and physical properties to achieve a broad range of specific surface free energies suitable for biosensing applications [31]. Since the fluid propagation in capillary driven microfluidic devices does not obey the wicking principle in porous paper-based media [32], this allows the flow of reagents to be regulated in a more precise manner with the possibility of introducing, for example, stop or delay flow valves to modulate time for analyte binding. In addition to fluid controls, plastic LFTs have a strong potential for scalable manufacturing. Plastic LFT chips with 2.5D geometries can readily be manufactured by conventional methods, such as injection molding (IM) [33], hot embossing [34], nanoimprint lithography (UV-NIL [35,36], and EC [37,38]), making them a better-suited vehicle than paper to construct more complex fluidic architectures with an ample fluid capacity often required by lab-on-a-chip applications. In the landscape of COVID-19 lateral flow tests, COVID-19 IgG xPOC (GENSPEED Biotech GmbH, Rainbach im Mühlkreis, Austria) is an example of a commercial IVD-certified LFT that takes advantage of the manufacturability and optical property of injection-molded plastic for quantitative multiparametric detection of SARS-CoV-2-specific IgG based on chemiluminescence. According to a recent study that cross-compared the performance of multiple COVID-19 serologic assays, this rapid point-of-care serological test exhibited a high sensitivity and specificity of 100% and 93%, respectively [39].

In the ecosystem of microfluidic device manufacturing, injection molding has long been the staple of chip production owing to its scalability and ability to realize 2.5D designs with precision and a considerable throughput [40,41]. Nevertheless, attention has recently been shifted to foil-based LFT devices that could also be fabricated with 2.5D structures, allowing us to explore alternative technologies that offer cost reduction in production. In light of this opportunity window, we leveraged R2R UV-NIL and EC imprinting technologies to address the rising demand for LFT for point-of-care testing applications. UV-NIL creates microfluidic structures on the carrier foil by using a stamp to pattern a photocurable resin, which is subsequently cross-linked under UV exposure (Figure 1B–D). The patterning of structures on a foil is achieved in a similar manner by EC [42,43], which uses molten thermoplastic instead of UV-curable resins (Figure 1E,F). The unique advantage of these two techniques resides in their capacity for high-volume, continuous production when implemented in the R2R system (Table 1). In the past, UV-NIL [44] and EC [45] were utilized for biosensing applications. Recently, Toren, et al. demonstrated a proof-of-concept for scalable biofunctionalization process by implementing a nucleic acid-based detection assay on mass-produced foil-based microfluidic chips [28,46]. These advancements highlighted the significance of R2R nanoimprint lithography as the enabling platform for the future LFT chip production.

In this article, we detail our continuous effort to improve upon the economy of scale of chip production while also demonstrating the clinical relevance of foil chips through quantitative chemiluminescence assay. To achieve this goal, we focus our effort on establishing two parallel manufacturing schemes that pivot UV-NIL and EC for the scalable production of foil chips where imprinting, non-contact surface biofunctionalization, and lamination are combined into seamless, high-throughput R2R manufacturing processes. We highlight the translational aspect of this work by describing the adaptation of the COVID-19 IgG xPOC assay from its injection molding system onto our foil-based LFT chips with minimal modifications. To our knowledge, our current investigation offers significant improvements concerning the extension of channel height [34] and the lamination method [28] of R2R-manufactured microfluidic devices, an achievement that further advances foil-based LFT (lab-on-a-foil) systems towards emerging applications in point-of-care testing.

## 2. Materials and Methods

### 2.1. Materials and Reagents

Unless otherwise stated, all reagents for surface biofunctionalization, proprietary reference solutions containing undisclosed amounts of SARS-CoV-2 neutralizing antibodies, and other measurement reagents for the COVID-19 IgG xPOC assay were provided by GENSPEED Biotech GmbH (Rainbach im Mühlkreis, Austria). Proprietary thiol-containing acrylate-based mr-BioNIL100SF_XPA UV-curable resin (referred to as “XPA” in this manuscript for brevity) was provided by micro resist technology GmbH (Berlin, Germany). Proprietary acrylate-based NILcure_M1 UV-curable resin and fluorinated anti-adhesion coating for the imprinting master were developed at JOANNEUM RESEARCH Forschungsgesellschaft mbH (Institute MATERIALS, Weiz, Austria). Polyethylene terephthalate (Melinex ST505 PET, DuPont Teijin Films) was purchased from Pütz Folien GmbH (Taunusstein, Germany). Cyclic olefin copolymer (COC) pellets were purchased from Topas Advanced Polymers GmbH (Oberhausen, Germany). ARflow 93025 pressure sensitive adhesive (PSA) foil was purchased from Adhesives Research (Limerick, Ireland).

### 2.2. Chip Designs and Simulations

The original 3D design of injection molded microfluidic test chip was adapted into corresponding 2.5D designs (Figure 1G) for UV-NIL and EC technologies in SolidWorks (version 2021, Waltham, MA, USA). The inlet and waste compartment were modified within the operational constraint of the portable GENSPEED analyzer equipped with programmable fluid injection capability and the technical limitations of R2R imprinting. Potential air entrapment caused by surface contact angle variations due to defects was initially interrogated with computational fluid dynamics (CFD) simulations using Ansys Fluent to determine the underlying conditions for bubble formation during the flow (Ansys, Canonsburg, PA, USA). The pressures at inlet and outlet were set to atmospheric pressure (101 kPa). Inlet accounts for the hydraulic losses (Hagen-Poiseuille) [47,48] coming from the measurement channel. The default contact angle of the channel (2 mm × 20 mm) was fixed at 55°. The contact angles of a defect area (1 mm × 1 mm) varied from 80° to 90°, 110°, and 160°. With the consideration for ultrasonic welding, the EC chip incorporated an additional 100 µm tall energy director structure that forms a wall around the sensing and waste channels for the purpose of bonding.

### 2.3. Surface Characterizations and Flow Rate Measurements

Channel geometry and cross-sectional images of the imprints were taken with scanning electron microscopy (SEM). Water contact angles of different materials were determined by the sessile drop method (DSA100, A. KRÜSS Optronic GmbH, Hamburg, Germany). Double bond conversions of acrylate resins after UV polymerization were determined by Fourier-transform infrared (FTIR) spectroscopy (Bruker, Billerica, MA, USA). Volumetric capillary flow rates of reagents used in the COVID-19 IgG xPOC were measured by dispensing each reagent according to the actual volume and sequence specified in Table S1 at the chip inlet and measuring the total time required for the fluid to be totally drawn into the chip’s sensing channel.

### 2.4. R2R Nanoimprinting

A nickel master stamp (280 mm × 629 mm) bearing the negative microfluidic structures was prototyped and fabricated by Temicon GmbH (Dortmund, Germany). The Ni master pre-treated with fluorinated anti-adhesion agent was wrapped around the imprinting cylinder to enable continuous UV imprinting, allowing a total of 36 LFT chips to be imprinted per revolution. A slot-die coater was used to apply liquid NILcure_M1 resin on a moving 125-µm thick PET carrier foil (Melinex ST505). UV imprinting was performed on the R2R UV-NIL replication pilot line at JR (Coatema modular system, Coatema Coating Machinery GmbH, Dormagen, Germany) with a web speed of 4 m/min. Photocuring was achieved with 100% UV power at 365 nm. (100% is equivalent to 14 W/cm².) The UV unit line pressure was set to 100 N/line. The quality of the imprint was inspected in real-time by the line camera (TELEDYNE E2V ELIIXA+ 16k, Rauscher GmbH, Olching, Germany) installed after the imprinting unit. R2R extrusion coating was achieved by imprinting COC on PS carrier foil through a similar process as that described in Okulova, et al. [43].

### 2.5. R2R Biofunctionalization

To demonstrate the feasibility of high-throughput biofunctionalization of the foil chips, we employed a non-contact piezoelectric microarray spotter customized for R2R operation (S12 sciFLEXARRAYER, Scienion AG, Berlin, Germany) as previously described [28]. To enable automated foil handling, the spotter is additionally equipped with external winding units (Sondermaschinen Oschersleben GmbH, Oschersleben Germany) for foil manipulation (Figure S1A). The winding unit is integrated with an in-line deionizer and sensors to ensure a precise foil alignment during movement. The web tension and foil rolling speed were set to constant 75 N/m and 5 m/min, respectively. In this setup, the foil was released from the roll with the structured side facing outward for the spotting nozzles to access the target surface. This sidedness was then reversed at the rewinding unit, such that the structured side was facing inward during wrapping to protect the immobilized probes from potential physical damage during transport for lamination.

In regard to the assay transfer, we increased the immobilization concentrations of receptor binding domain (RBD), nucleocapsid protein (NP) and Spike protein (SP) probes from specifications used for the GENSPEED’s chip. In compliance with the original method, immobilization in R2R mode was achieved by passive adsorption in which the capture probes were dispensed and allowed to incubate on the substrate surface at an ambient temperature under 40% relative humidity, following the loop process schematized in Figure S1B. All SARS-CoV-2 antigen probes were immobilized at the final concentration of 0.25 mg/mL. Biotinylated anti-human IgG (positive control) was immobilized at 0.5 μg/mL. All probes were spotted with a final volume of 336 nL on the specific positions along the length of the sensing channel for multiplex chemiluminescence readouts (Figure S1C). To mitigate the overspreading of probe solutions, spotting was divided into two rounds of 168 nL. The reagents spotted during the first round were allowed to dry (approximately 1 min) before the second round of spotting. These positions spatially correlate to the “pixel” coordinates of the photodiode array on the portable GENSPEED analyzer.

### 2.6. Lamination and Singulation

After surface biofunctionalization, UV-NIL imprints (Figure 1H,I) were laminated under a similar R2R UV-curing process described in Section 2.4. The cover foil (Melinex ST505) was pre-treated with R2R atmospheric plasma (corona) at 1 kW power. UV lamination utilized gravure coating to apply a thin liquid XPA resin layer on the cover foil. Bonding was accomplished through the combination of using the addition of a counter pressure roller and UV irradiation (14 W/cm^2^) at a speed of 1 m/min.

EC chips containing the energy director structure (Figure 1J,K) were ultrasonically welded to a flat sheet of COC cover foil. The cover foil was activated with oxygen plasma for 5 min to render the surface hydrophilic prior to ultrasonic (US) welding. A titanium sonotrode with a frequency and a lambda ½ amplitude set to 20 kHz and 25 µm, respectively, was used for welding through two consecutive forces. Force one (applied first) was set to 550 N. Force two (applied after the delay time of 0.07 s) was set to 750 N. The total weld time was limited to 0.145 s. After the application of the US energy, a hold time of 1 s with a force of 750 N was applied to cool the sample down while compressed. A sonotrode was customized to operate effectively with 2.5D structures regardless of energy director designs. This customization allowed for the iterative optimizations of energy director structures to be carried out during development. Note that ultrasonic welding in the present setup was not configured for the R2R production. Thus, lamination of EC chips was still achieved in a scale of tens of chips per batch. During the initial testing, pressure-sensitive adhesive (PSA) was used as a hydrophilic cover foil, allowing rapid chip lamination to be accomplished in a peel-and-stick manner on both UV-NIL and EC chip variants.

In the current development, venting hole drilling, inlet cutting, and chip singulation were achieved by a non-R2R laser cutting operation using a CO_2_ laser source. Hole drilling on UV-NIL chips was performed as a back-end process that took place after the imprint was fully laminated. Conversely, this hole drilling step of EC chips was executed directly after imprinting. Chip singulation by laser cutting was carried out as a back-end process for both types of foil chips.

### 2.7. On-Chip SARS-CoV-2 Antibody Quantitation via Chemiluminescence

Biofunctionalized UV-NIL and EC chips were evaluated on the GENSPEED analyzer (R1 device) that measures chemiluminescence signals emitted from the chip along the sensing channel (Figure 1G). The multistep measurement protocol of the COVID-19 IgG xPOC assay was modified to accommodate the reduced fluid capacity of the foil chips compared to the standard injection molded (IM) chips (Table S1). Reagents were sequentially flowed across the chip based on capillary force and allowed to incubate on the surface. To evaluate the foil chips in our current investigation, two proprietary solutions containing RBD-, NP-, and SP-specific IgG antibodies were used as surrogates for a fingerprick blood sample (20 µL) in the actual assay, allowing us to compare the signal profiles of UV-NIL and EC chips against injection molded (IM) standard. It is crucial to note that we still relied on using GENSPEED’s proprietary reference solutions for testing the foil chips at this stage of development to determine the preliminary suitability of our R2R imprinting technology candidates. These reference solutions contain undisclosed amounts of each antibody analyte. Therefore, the standard evaluations of biosensors concerning sensitivity, specificity, and the limit of detection will be performed in our forthcoming investigation once the manufacturing process is finalized.

### 2.8. Data Collection and Statistical Analysis

Chemiluminescence intensity emitted from the test chip was detected by an array of 30 photodiodes over a fixed duration of 2:30 min. Pixel-specific data were processed by a custom-developed MATLAB analysis tool (version 2015a, MathWorks, MA, USA) that parses the cumulative intensity corresponding to each position and applies the Gaussian fitting to obtain the final readout values. Numerical data were plotted and analyzed by Origin Pro (OriginLab, MA, USA). Statistical differences of the means between test groups were determined by two-way student’s *t*-test with alpha = 0.05.

## 3. Results and Discussion

### 3.1. Chip Design and Simulations

**Figure 2:**
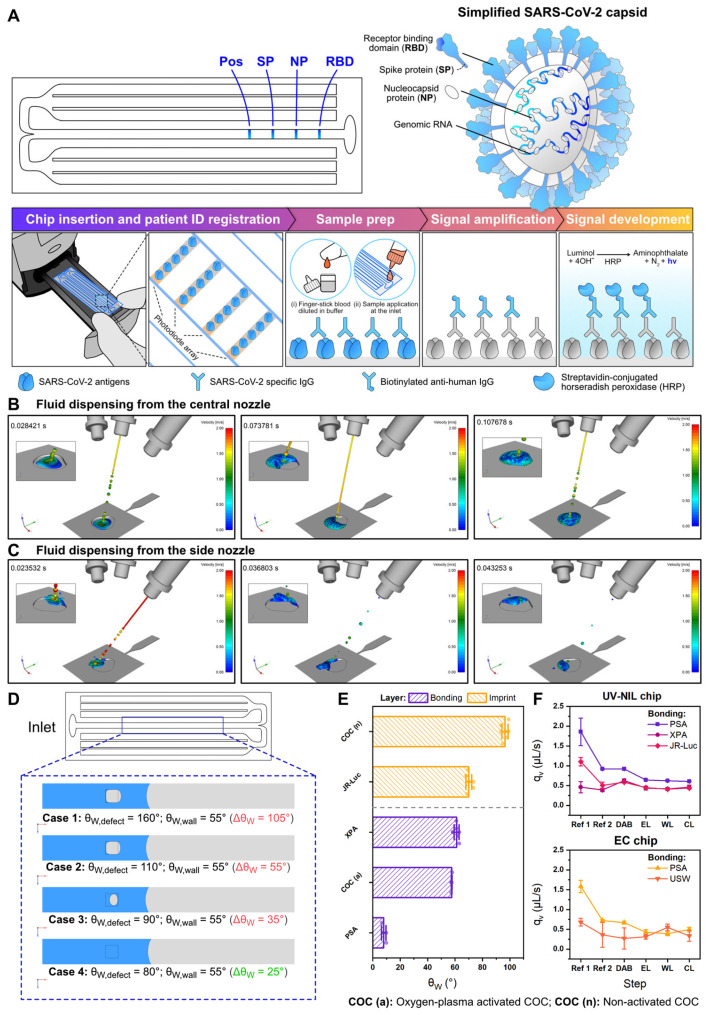
(**A**) Schematic illustration of the surface biofunctionalization with SARS-CoV-2 antigen probes, and the measurement process of the multiplex COVID-19 IgG xPOC µ-ELISA assay. (**B**) Time-lapsed simulations from 10 µL fluid injection from the central nozzle of the GENSPEED reader set at the default 2 cm offset above the chip into the chip. (**C**) Time-lapsed simulations from 2 µL fluid injection from the side nozzle of the GENSPEED reader set at the default tilting angle and a 2 cm offset above the chip into the chip. (**D**) Preliminary investigation by computational fluid dynamics (CFM) simulations of surface inhomogeneity to determine the threshold of the water contact angle (θ_W_) differences between channel defects and the wall for maintaining bubble-free capillary flow. Four different values for θ_W,defect_ (in the range 70–160°) were investigated, as compared to θ_W,wall_ = 55° (**E**) Water contact angles of different materials (resins and plastic foils) used to assemble UV-NIL and EC chips. The materials are categorized on the diagram based on their functionalities. UV-NIL imprints (NIL_cure_M1) can be laminated with pressure sensitive adhesive (PSA) and UV-curable resin (XPA). EC imprints (non-activated COC or COC(n)) can be laminated with O_2_-plasma activated COC (COC(a)) via ultrasonic welding, and also by PSA. Note that the water contact angles of COC(a) were measured immediately after plasma activation. (**F**) Breakdowns of stepwise volumetric flow rates (q_v_) of foil chips that are laminated with different approaches for COVID-19 IgG xPOC reagents, which are reference solution 1 (Ref 1), reference solution 2 (Ref 2), detection antibody (DAB), enzyme solution (EL), wash solution (WL), and chemiluminescence developer solution (CL).

The original GENSPEED IM chip was redesigned to fulfill the specifications of R2R fabrications whose achievable channel depths are lower than injection molding’s. This practical restriction imposed a limitation on the total fluid capacity that the foil chips could hold. Its 2.5D analogs for UV-NIL and EC chips shared a common layout of having a relatively large inlet concerning the chip’s width and six waste channels that branch out on both sides of the chip to utilize the entire chip footprint (Figure 1G). Foil chips were also designed with minimal sharp edges and corners (Figure 1H) to avoid defects during R2R imprinting [36]. The standard IM chip (GENSPEED) has two different channel depths, which are 100 µm and 600 µm in the sensing channel and waste compartment, respectively. This translates to the total fluid capacity of 200 µL. UV-NIL foil chips were imprinted at the uniform channel depth of 125 µm (Figure 1I). As a result, the total fluid capacity of a UV-NIL chip is approximately 130 µL after lamination. Given that the EC chip could not undergo the same process for lamination, its design was augmented with an additional layer of 100 µm high-energy director wall that envelopes the sensing and waste channels for bonding with ultrasonic welding (Figure 1J,K). This design limited the maximum channel depth of EC chips to 100 µm to permit proper sealing of the chips, resulting in a total fluid capacity of 80–90 µL. This significant reduction in the total fluid capacities necessitated the optimization of the assay protocol (Figure 2A and Table S1), with respect to, e.g., reagent volumes and incubation time, to ensure adequate mass transport to the sensor surface that influences the optical signals.

A large oval inlet of the foil chips in comparison to the original 3D barrel-shaped design was flattened and widened to retain its ability to hold the reagent drop. Fluid flow that took place spontaneously via capillary force was initiated by the dispensing of reagent at the inlet. In the commercial setup, this process would occur inside an automated device that dispenses multiple drops of reagents at specified volumes from a piezoelectric reagent cartridge. Therefore, the inlet area would have to be sufficiently large to accommodate the buildup of liquid up to 30 μL. During the design phase, we simulated the dispensing process from the central (Figure 2B) and side (Figure 2C) nozzles. We found that the default inlet position and the 2 cm inlet-to-nozzle offset, both of which are fixed parameters required by the reader, were optimal for reagent dispensing. A lack of sharp corners at the inlet was also critical to a proper drop formation as it helped guiding the fluid into the sensing channel without leaving residual around the edges. Using CFD simulations, we also interrogated the potential scenarios for air entrapment in the flow channel. This investigation took into account a defect area characterized by four different water contact angles (θ_W, defect_ = 80°, 90°, 110°, 160°), each being greater than that of the imprint (θ_W, wall_ = 55°) (Figure 2D). The computational outcomes revealed that a discrepancy of less than 30° was sufficient to induce an air pocket within the channel, the degree of which is directly proportional to the θ_W_ differences. This preliminary discovery stressed the importance of maintaining surface homogeneity during fabrication to prevent the entrapment of air. Once formed within the microfluidic channels, air pockets could interfere with mass transfer, fluid flow, or optical measurements, ultimately leading to false recorded signals.

### 3.2. Gauging the Material Compatibility of Foil Chips for Assay Implementation

In an effort to directly transfer the multiplex COVID-19 IgG xPOC assay onto foil chips, we first tested the feasibility of our approach by performing an assay on UV-NIL and EC chips prepared in a batch scale, i.e., without engaging full R2R biofunctionalization and R2R lamination. This scale of imprint preparation, termed “non-R2R”, exploits hydrophilic PSA to achieve rapid batch-scale lamination on both types of foil substrates. Since the imprints are available at different water contact angles (Figure 2E), this bonding method warranted their direct comparison under a similar flow condition, which occurred initially at 1.5–2 μL/s (Figure 2F). Our present study emulated GENSPEED’s production to harness passive adsorption as a principal R2R-compliant method for the immobilization of protein-based probes on both UV-NIL and EC substrates. This instantaneous surface immobilization strategy is vital for a cost-effective assay transfer and upscaling on the R2R production systems. Being independent of covalent immobilization techniques also sets our approach free from relying on surface chemical modifications that are difficult to execute in a well-controlled manner in R2R settings. While passive immobilization of proteins on thermoplastic or polymeric surfaces has been vastly demonstrated in the literature [49], this investigation allowed us to investigate the feasibility of protein adsorption on the resin-coated (UV-NIL) surface, which has never been reported elsewhere.

**Figure 3:**
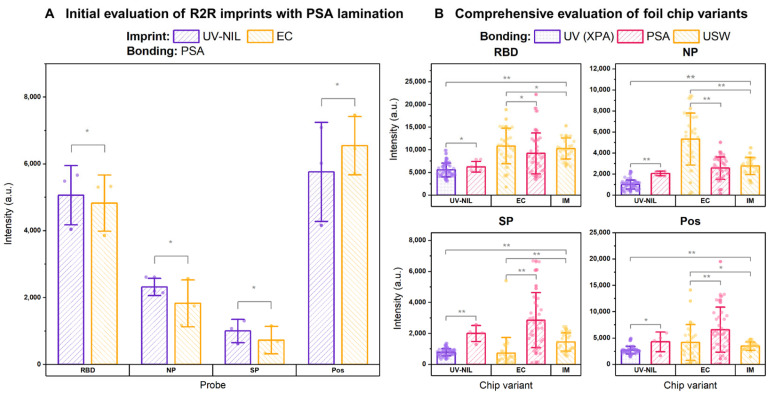
(**A**) Preliminary feasibility testing of UV-NIL and EC imprints as suitable candidates for assay implementation. To enable rapid lamination, we used hydrophilic PSA as a bonding layer to form the cover foil for both types of imprints (N = 3 for each variant), which initially showed comparable chemiluminescence across all probes. (**B**) Comprehensive evaluations of foil chip performance and the effect of lamination strategies with respect to GENSPEED’s IM standard chips (N = 25). In the UV-NIL group, PSA-bonded chips (N = 4) exhibited higher signals compared to UV-laminated variants (N = 51), possibly due to UV-induced damages (UV-dose: 14 W/cm^2^). In the EC group, ultrasonically welded (USW) chips (N = 28) showed higher signals in RBD and NP than PSA-laminated variants (N = 33), suggesting the effect of a slower flow rate that permitted a better mass transport to the sensor’s surface. (* denotes *p* > 0.05, ** denotes *p* < 0.05).

For the interest of COVID-19 serology test, three structural antigens were used to discriminate the level of SARS-CoV-2 specific IgG antibodies induced either by infection or vaccination. Since the spike protein (SP) was exploited as the target for most early vaccines [50], the presence of IgG molecules against it in the test samples indicates long-term adaptive immunity generated from vaccination, whilst that corresponding to NP or RBD suggests a history of past infections [51]. After identifying the optimal parameter for preparing the UV-NIL imprint and evaluating its stability in storage (Figure S2A–C), we compared the influences of foil materials from the two R2R imprinting methods on the chemiluminescence signals measured with the modified protocols (UV-NIL-Short and EC-Short in Table S1). The profile of chemiluminescence responses observed on the EC chips followed the same fashion as that observed on UV-NIL chips, with RBD contributing to the highest signals (Figure 3A). This initial finding provided basic evidence that supported the suitability of our imprint materials as candidates for biochemical assay implementation. Since the main goal of our present study was to highlight the R2R manufacturing techniques and investigate their feasibility for a lab-on-a-chip application, we adhered to the original passive immobilization strategy to enable a direct comparison between the foil chips and the standard IM chips without any molecular modifications of the capture probes or buffer to modulate the signals.

### 3.3. R2R Biofunctionalization: A Critical Component in High-Throughput R2R Fabrication Ecosystem

With promising results in the initial assay implementation, we streamlined the surface biofunctionalization to seamlessly integrate this crucial operation with the other R2R processes, e.g., imprinting and lamination. Given that passive adsorption requires incubation and dehydration, we set a canonical R2R spotting operation to be the recurring cycle of stop-and-go processes (Figure S1B). The foil is threaded into the microarray spotter by coordinated movements of the winder and rewinder to position the spotting targets (i.e., sensor chips) on a vacuum stage. Once the foil is in place, the stage will be raised while activating the vacuum suction to form a better contact with the foil, initiating the spotting process as performed for the batch production. To further locate and orient the targets with micron-scale precision, each chip contained two diamond-shaped fiducial features (Figure S1C), which served as reference points that could be automatically recognized by the spotter’s machine vision. These two fiducials are located diagonally across the sensing channel from each other to provide the angular correction for spotting.

While a simple printing pattern employed in this work is conceptually achievable with a striper commonly used for paper-based LFT functionalization, the 2.5D topology of our imprinting substrates imposed a physical barrier for a striper to function properly. Our utilization of a robust contactless piezoelectric dispenser not only reduces reagent consumption for manufacturing but also offers a capability for intricate array designs that could be implemented at the later stages of chip development. To improve the speed of dispensing, we dedicated one piezoelectric nozzle to each antigen, thereby achieving the total active time for spotting all probes of less than 10 s/chip. Once all targets had been functionalized, the foil was released from the vacuum stage and wound in preparation for the subsequent R2R lamination step and back-end process. This current configuration of our R2R biofunctionalization machinery represents a critical component that enables high-scale surface functionalization while preserving the integrity of the entire manufacturing system that brands itself around seamless handling. Thus, our effort in establishing and optimizing the high-throughput surface biofunctionalization is instrumental to the future advent of multiplex μ-ELISA LTFs or microfluidic devices where the versatility of the dispensing system can play a pivotal role in promoting their evolution towards miniaturization.

### 3.4. Comprehensive Evaluation of R2R Foil Chips with SARS-CoV-2 Antibody Detection

To evaluate the performance of the foil chips, we performed a comprehensive cross-evaluation of UV-NIL chips (laminated with R2R UV lamination and PSA control) and EC chips (laminated with ultrasonic welding and PSA control) following a modified protocol with increased incubation time (UV-NIL-Long and EC-Long in Table S1). The overall chemiluminescence signals obtained from this scaled evaluation were in accordance with preliminary testing for both types of substrates. The effect of direct UV radiation on the immobilized protein probes during lamination of UV-NIL chips was determined by comparing the chemiluminescence signals of R2R UV-laminated chips against the PSA controls that were not exposed to UV. Despite a relatively faster fluid flow rate on PSA-bonded UV-NIL chips (Figure 2F), their NP and SP signals were two folds higher than those of the R2R UV-laminated variants (Figure 3B). This result suggested that subjecting the capture protein probes under UV exposure (14 W/cm² at 1 m/min) during R2R lamination may account for the loss of signals, as UV potentially might harm protein probes through multiple UV-induced mechanisms, most notably, photo-oxidation [52]. Since the signal strengths of the sandwich ELISA assay directly depend on the affinity between capture probes and their target analytes, the ability of the target IgG to recognize the probe could be compromised by structural alterations that affect molecular interactions [53]. 

However, there are a number of other factors that can decrease the signals of the R2R UV-laminated chips, like traces of contaminants and channel obstruction. We demonstrated in further experiments (described in the Supplementary Information in details) that by keeping the UV dose constant at 14 W/cm² at 1 m/min, we could still improve the signals of the capture probes, notably, RBD, by nearly threefold. This optimization was accomplished through a combination of two measures: First, by revising the method for R2R lamination that utilized the NIL_cure_M1 resin instead of XPA, thereby changing the flow rate towards the value observed for the PSA tape lamination (Figure S3). And second, by characterizing the performance of UV-NIL chips in large-scale imprinting (100 m) (Figure S4) that revealed the detrimental influence of the optional F-based anti-sticking coating on the Ni stamp. Through the long imprinting runs, we could also demonstrate the stability of UV-NIL chips over the course of 4 weeks in storage (Figure S5).

As an alternative approach, R2R EC chips were fabricated by directly imprinting thermoplastic COC on the carrier foil, requiring a different technique for bonding from UV-NIL chips. The lamination process of EC chips employed in our investigation used a scalable R2R-compliant ultrasonic welding to selectively melt and fuse the energy director on the imprint to the COC cover foil. Since we opted to preserve the native surface property of COC on the imprint foil for biofunctionalization, the cover foil was treated with oxygen plasma to render itself hydrophilic to permit capillary flow. The effect of this lamination approach was evaluated with a direct comparison of chemiluminescence signals of the EC chips closed with PSA. In comparison to the non-optimized UV-NIL counterparts, EC chips exhibited a similar pattern of signals involving a dominating presence of RBD with respect to the other probes. However, their RBD and NP signals were approximately 2- and 5-fold greater than the average signals measured on UV-NIL chips while their SP signals were comparable (Figure 3B).

When comparing the lamination techniques of EC chips, we observed that ultrasonic welding also offered superior signals of RBD and NP probes over PSA, whose averages were on par with IM benchmark (Figure 3B). Since lateral flow took place at a significantly slower rate on ultrasonically welded chips than on the PSA-laminated variants, the moderate initial flow rate of ca. 0.7 μL/s could be a predominant factor that promotes better mass transport to the surface in contrast to the flow rate of ca. 1.6 μL/s enabled by PSA bonding (Figure 2F). In contrast to the other two probes, the SP and positive control of ultrasonically welded chips both exhibited lower signals compared to those of PSA variants and the optimized UV-NIL chips in Figure S4. The paradox observed here raised a concern that this unexpected deterioration of the SP and Pos signals was attributed to other factors beyond biofunctionalization. As a result, a further investigation of the EC chip is required in the future to determine the root cause of this issue.

### 3.5. Towards Sustainable Manufacturing of the Foil Chips

From the perspective of process developers, we hope to see a growing interest from the manufacturers to reap the benefits of an unparalleled economy of scale and speed of surface biofunctionalization endowed by the configurable R2R ecosystems. Our effort in implementing the COVID-19 serologic assay transferred from conventional injection molding to high-throughput R2R-fabricated chips serves as a milestone that unlocks a new possibility for other multiplex assays to be reformatted and miniaturized into LFT. To improve the turnaround time of on-chip analysis, we have shown that the foil chips could still generate comparable signals after introducing a measurement protocol requiring a shorter incubation time (Figure S6) to align them with the operational standard of point-of-care testing. Although the demand for COVID-19 testing is fading as a standalone test, novel LFT assays will still hold their value and significance in healthcare. Being able to be deployed in decentralized settings, these assays will continue to play a fundamental role in combating existing public health challenges, such as detecting microbial drug resistance (“silent pandemic”) and surveilling potential pandemic pathogens (PPP) [4]. An increased demand and variety of emerging assay options for single-use LFT devices [54] also critically calls for better plastic waste management in medical diagnosis [55], which currently requires disinfection through incineration.

In light of sustainability, a transition from injection molding to R2R imprinting accounts for a decrease of nearly four times the quantity of plastic when considering the weight difference between IM (ca. 3 g) and foil chips (ca. 0.8 g). Additionally, the PET and COC utilized in this work as raw materials for foil chips are both known for being recyclable materials. Although the availability of commercial recycled PET in the foil format is currently highly limited, we believe that the utility of foil-based chips demonstrated here will galvanize the development of high-quality recycled PET foil products that can be used for R2R imprinting. Once available, the use of recycled PET and COC could achieve a significant reduction in the new plastic required to fabricate LFT chips. While a more sustainable future of the end-of-life policies is still up for an extensive discussion and policy making, our attempt in assay transferring to a foil-based platform could also be regarded as a pivotal initiative to address plastic consumption and promote the use of recyclable materials in the manufacturing process of novel polymer-based LFT devices via R2R technologies.

## 4. Conclusions

In response to the increasing global need for point-of-care testing, we demonstrated how the marriage of R2R imprinting, R2R biofunctionalization, and R2R lamination processes were harnessed to streamline and upscale foil-based LFT fabrications. Our work serves as a foundation for upcoming developments that aim to construct a more seamless and self-contained ecosystem for foil chip production. The transferring of model serologic assay, highlighted in this work through comparable chemiluminescence signals between chip variants, is a testament to the adaptability of foil chips to accommodate multistep μ-ELISA assays. With the rising significance of LFT devices in addressing both existing and emerging global health challenges, we believe that the untapped power of the R2R fabrication technology will revolutionize how emerging foil-based LFT devices are manufactured and processed after use, balancing the testing demand with an important awareness of a circular economy in the diagnostics industry.

## 5. Outlook

Our current data strongly demonstrates the potential of foil chips as a future vessel for biochemical assay implementation. However, we acknowledge the limited investigation into the sensoric performance of the foil chips due to the scope of this study. Our aim was to provide a preliminary direct comparison between GENSPEED’s original injection-molded chips and the foil-based counterparts using GENSPEED’s proprietary references. Given this limitation, we are actively working on further optimizations of the biofunctionalization and measurement protocols to enhance signal quality. This effort involves extensive coordination among multiple partners in the manufacturing value chain to achieve the optimal version of the foil chips. Once the preparation details of the foil chips are finalized within the manufacturing process, we plan to thoroughly characterize the sensor performance. This includes determining the limit of detection and quantification in dilution series of analytes and performing validation measurements with cryo-preserved plasma samples to determine the sensitivity and specificity. Although the demand for COVID-19 testing has decreased overtime, we are confident that the knowledge generated from this study will serve as a valuable model for the future development and implementation of micro-ELISA assays on the foil chip format to facilitate quantitative antigen testing, such as cardiac disease markers, etc., at the point of care.

## Figures and Tables

**Table 1:** The following table summarizes the main advantages and drawbacks of the applied techniques for polymer replication of microfluidic structures compared to injection molding. Calculations are performed for chips in microscope slide format, 25 × 75 mm^2^.

Property	Injection Molding	Roll-to-Roll Imprinting	
Fluidic patterns:			
**Typical number of chips per working tool**	6	36 and more **Advantage:** More design variations can be placed on same working tool for intensive parallel testing.	
**Throughput, typical numbers for replicated chip patterns/min**	12	120 and more **Advantage:** Minimum of 10-fold increase of process throughput is possible.	
Post processing:			
**Implementation of bioprinting and lamination processes**	**Disadvantage:** Individual chip handling required (manual or robot-based).	Processing of large-area-sheets (e.g., with 24 chips and more) or roll-to-roll processes. **Advantage:** increased throughput due to continuous chip processing on same substrate.	
Materials:			
		**UV nano imprint lithography (UV NIL)**	**Extrusion Coating**
**Processed material**	Thermoplastics. Advantage: use of known and certified polymers. Disadvantage: limited material tunability.	UV curable materials. **Advantage**: wide tunability of surface, mechanical and optical properties, etc. The main advantage here is material intrinsic hydrophilicity. **Disadvantage:** Material verification might be required (dependent on application).	Thermoplastics. **Advantage:** use of known and certified polymers. **Disadvantage:** limited material tunability.
**Material and energy demand**	Thick, rigid polymer elements. High process pressure and temperature.	Thin polymer foil chips, reduced material consumption.	
		Process without active heating, and hence, low energy consumption.	Lower process pressure than in injection molding.

## Data Availability

Public data are available online on an Open Science Framework (OSF) platform via https://osf.io/pqrjc/?view_only=709af66c44bc4ac99c2cb827334b1cf9 (accessed on 1 March 2025).

## Supplementary Materials

The following supporting information can be downloaded at: https://www.mdpi.com/article/10.3390/bios15040229/s1, Figure S1: Instrumental setup for the R2R biofunctionalization unit. (A) Breakdowns of R2R biofunctionalization unit: (1) a winder for the R2R UV-NIL imprinted foil microfluidics, (2) S12 medium-scale microarray spotter, (3) a UV-curing unit, and (4) a rewinder. (B) Canonical R2R surface functionalization process illustrating the coordinated commands of stage and foil movement. The precise alignment of spotting target (i.e., sensing channel of the microfluidic chips) can be achieved by fiducial recognition by the spotter’s integrated machine vision. Quality and drop volume of reagent droplets are closely tracked throughout the duration of spotting to minimize a variation of immobilized detection probes across the target. (C) Magnified image of a foil chip layout highlighting the two fiducial structures utilized for target alignment. Figure S2: Optimization of UV curing dose for R2R UV-NIL. (A) Optimization of the UV photocuring power for R2R UV-NIL imprint prepared with NIL_cure_M1 resin (N = 3 for each curing dose). With the exception of the SP probe, we observed a consistent trend of rising chemiluminescence signals as the curing doses increased. Therefore, UV-NIL imprints were prepared with 100% curing power (14 W/cm^2^) in the subsequent investigations. (B) Determination of double bond conversion (DBC) rates for UV imprints prepared at different UV photocuring doses. A higher percentage of conversion is observed at a higher UV power exposure, affecting the stability of the imprints. (C) Stability of UV imprints prepared between high (fully-cured) and moderate (semi-cured) photocuring doses. While the water contact angles of fully-cured imprint remained relatively stable over a period of 80 days in ambient storage, the surface energies of a semi-cured imprint fluctuated, resulting in the decrease of water contact angle by larger than 15°. Figure S3: Revised method for R2R UV-NIL lamination. (A) Peeling strength of UV-NIL chips prepared with two photocurable resins, NIL_cure_M1 (new approach) and XPA (former approach), which exhibits a higher peeling strength. However, due to the surface energy mismatch of XPA with the imprinting resin (NIL_cure_M1), we re-optimized the bonding process to permit stable chip lamination while still complying to the established R2R setup and retaining a desired flow characteristic. (B) Volumetric flow rate of UV-NIL chips achieved with NIL_cure_M1 as a laminating agent (▲) in comparison to the previous variants laminated with PSA (■) and the XPA resin (●). Figure S4: Evaluation of chemiluminescence signals on 100 m of UV-NIL foil. (A) Evaluation of the effect of large-scale R2R UV imprinting and the change of UV bonding resin from XPA to NIL_cure_M1 on chemiluminescence outcomes (N = 12 for each position). All COVID-19 test probes exhibited significant signal improvements from the starting (meter 0) to the end (meter 97) positions with comparable signals between meters 25 and 75. (B) Water contact angles of the imprint surface at different imprinting length indices exhibited no significant variations at different positions. (C) Atomic percentages of trace elements on the imprint surface at different imprinting length indices. The decreasing fraction of fluorine suggests the depletion of fluorinated species over the course of imprinting that spanned 100 m in total. The potential source of F is anti-adhesion coating that was applied on the Ni master prior to imprinting to assist with demolding. Given the universal increases of chemiluminescence towards high position indices, the presence of F, albeit scarce, resonated with the reduction of probe’s activities. (* denotes *p* > 0.05, ** denotes *p* < 0.05). Figure S5: Stability of UV-NIL chips in storage. Stability of PSA-laminated UV-NIL chips in 4 weeks of storage at 4 °C showing consistent signal developments on all probes (N = 12 for each time point). The imprint was selected from the middle position (meter 50–51) of the 100-m R2R imprint (see Evaluation of chemiluminescence signals on 100 m of UV-NIL foil). (* denotes *p* > 0.05, ** denotes *p* < 0.05). Figure S6: Time optimization for COVID-19 IgG measurement on the foil chips. Time optimization of the adjusted measurement protocol for UV-NIL (N = 4) and EC (N = 3) chips laminated with pressure-sensitive adhesive (PSA). Time-optimized measurement could be completed with a shorter total incubation, which brings the on-chip analysis time to under 30 min. (* denotes *p* > 0.05, ** denotes *p* < 0.05). Figure S7: Visualization of liquid flow in microfluidic chip geometry. Liquid flow in the microfluidic chip geometry: (A) After the first pipetting, the liquid in the chip inlet begins to be drawn into the reaction channel by capillary action. (B) Once the inlet is emptied, a second dispensing is required for the continuation of liquid flow. (C) and (D) The sample channel is completely filled, and the capillary pump reservoirs are in the process of filling. Table S1: Measurement protocols for foil chips. Both variants of the foil chips have significantly lower fluid capacities than the standard IM chip from GENSPEED. Therefore, we adjusted the measurement protocol to enable on-chip chemiluminescence detection of the target IgG samples (reference solutions). For each foil chip variant, the measurement was performed with the reagent volumes specified in the highlighted columns. To compensate for smaller volumes used, longer incubation time (Long) was introduced to improve the signal development. However, we later demonstrated that a shorter incubation time (Opt) could also be used to achieve comparable signals under less total assay time. Table S2: Evaluation of residual elements at different position indices of a large-scale R2R UV imprint by X-ray photoelectron spectroscopy (XPS) analysis. Trace elements on the imprint surface were determined by X-ray photoelectron spectroscopy (XPS) by performing survey scans (high intensity-low resolution) of the whole spectral range in combination with highly prolonged integration time on specific positions. The detailed scans of the main element peaks with higher resolution but lower intensity were used for the peak fitting and data processing.
