# Supplementary material for: Translation of COVID-19 Serology Test on Foil-Based Lateral Flow Chips: A Journey from Injection Molding to Scalable Roll-to-Roll Nanoimprint Lithography
Source: Biosensors (Basel). 2025 Apr 4;15(4):229. doi: 10.3390/bios15040229 (PMC12024825; doi:10.3390/bios15040229)
Supplement: Supplementary file 1 [file biosensors-15-00229-s001.zip › biosensors-3401671-supplementary.pdf]

Supplementary Information

Translation of COVID-19 serology test on foil-based lateral flow chips: a journey from injection molding to scalable roll-to-roll nanoimprint lithography

Pakapreud Khumwan[1,*], Stephan Ruttloff[1], Johannes Götz[1], Dieter Nees[1], Conor O'Sullivan[2], Alvaro Conde[3], Mirko Lohse[4], Christian Wolf[1], Nastasia Okulova[2], Janine Brommert[5], Richard Benauer[6], Ingo Katzmayr[7], Nikolaus Ladenhauf[7], Wilfred Weigel[8], Maciej Skolimowski[3,9], Max Sonnleitner[7], Martin Smolka[1], Anja Haase[1], Barbara Stadlober[1], and Jan Hesse[1,9,‡]

[1]JOANNEUM RESEARCH MATERIALS, Institute for Sensors, Photonics and Manufacturing Technologies, Franz-Pichler-Strasse 30, 8160 Weiz, Austria
[2]Inmold A/S, Teglbuen 10, 2990 Nivå, Denmark
[3]Micronit B.V., Colosseum 15, 7521 PV Enschede, The Netherlands
[4]Micro resist technology GmbH, Koepenicker Strasse 325, 12555 Berlin, Germany
[5]Temicon GmbH, Konrad-Adenauer-Allee 11, 44263 Dortmund, Germany
[6]Bionic surface technologies GmbH, Liebenauer Hauptstrasse 2-6, 8041 Graz, Austria
[7]GENSPEED Biotech GmbH, Gewerbepark 2, 4261 Rainbach im Mühlkreis, Austria
[8]Scienion GmbH, Wagner-Régeny-Strasse 15, 12489 Berlin, Germany
[9]Microfluidics Innovation Hub, Franz-Pichler-Strasse 30, 8160 Weiz, Austria
*Present affiliation: National Center for Genetic Engineering and Biotechnology (BIOTEC), 113 Phahonyothin Rd., Khlong Nueng, Khlong Luang, Pathum Thani, 12120, Thailand

‡To whom correspondence should be addressed: Jan Hesse (jan.hesse@joanneum.at)

Supplementary figures

Figure S1. Instrumental setup for R2R biofunctionalization process 2
Figure S2. Optimization of UV curing power for UV-NIL imprint 3
Figure S3. Characterizations of UV-NIL chips laminated with NIL_cure_M1. 4
Figure S4. Effect of large-scale UV imprinting on chemiluminescence outcomes 6
Figure S5. Stability of PSA-laminated UV-NIL chips in storage 7
Figure S6. Time optimization of Covid-19 IgG detection on the foil chips 8
Figure S7. Visualization of liquid flow in microfluidic chip geometry.......... 9

Supplementary tables

Table S1. Measurement protocols for foil chips 10
Table S2. XPS analysis of UV-NIL imprints 11

S1. Instrumental setup for the R2R biofunctionalization unit

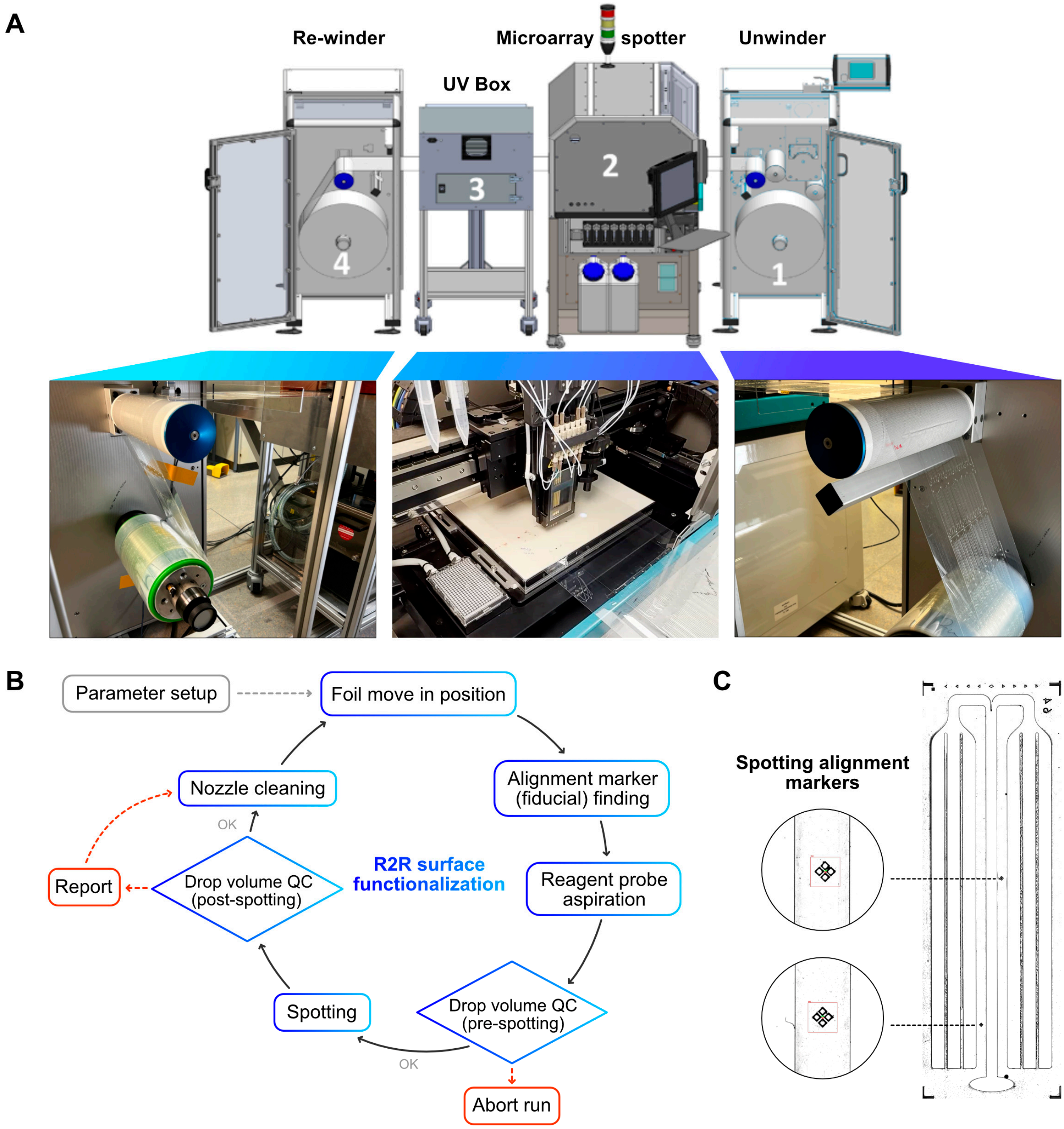

Figure S1 (**A**) Breakdowns of R2R biofunctionalization unit: (1) a winder for the R2R UV-NIL imprinted foil microfluidics, (2) S12 medium-scale microarray spotter, (3) a UV-curing unit, and (4) a rewinder. (**B**) Canonical R2R surface functionalization process illustrating the coordinated commands of stage and foil movement. The precise alignment of spotting target (i.e., sensing channel of the microfluidic chips) can be achieved by fiducial recognition by the spotter's integrated machine vision. Quality and drop volume of reagent droplets are closely tracked throughout the duration of spotting to minimize a variation of immobilized detection probes across the target. (**C**) Magnified image of a foil chip layout highlighting the two fiducial structures utilized for target alignment.

S2. Optimization of UV curing dose for R2R UV-NIL

We investigated the effect of R2R UV curing of UV-NIL imprints ranging from 70% to 100% power (10-14 W/cm^2) on the functionality of immobilized probes, following the modified measurement protocol (UV-NIL-Short in Table S1). Overall, the chemiluminescence signals across the curing doses slightly increased as we elevated the UV photocuring doses (Fig. S2 A). We observed highest average signals of RBD and NP at 100% curing power. This effect was reversed in the SP probe, where the highest signal is observed at 80% curing power and gradually decreased as the power rose. At higher photocuring dosages, we expected the imprints to also undergo greater double bond conversion (DBC) (Fig. S2 B), making them more stable in ambient light and humidity compared to under(semi)-cured imprints whose surface wettability could vary in storage (Fig. S2 C). Therefore, 100% curing power was later used in the preparation of R2R UV imprint for later testing.

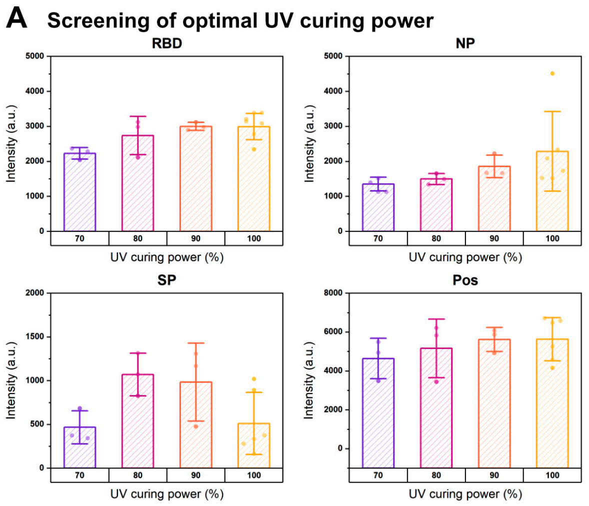

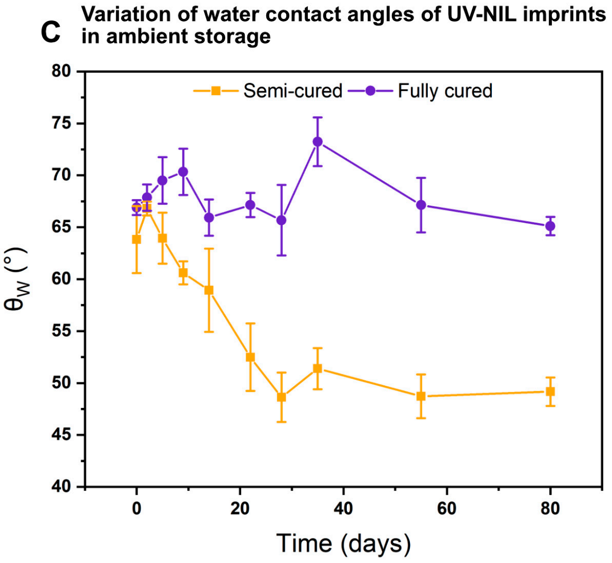

B **Double bond conversion (DBC) of acrylate resins**

Resin	C=O	=CH$_2$	Conversion
Imprints			
XPA R2R (Batch 1)	7.136	0.001	100%
XPA R2R (Batch 2)	11.441	0.127	94%
XPA non-R2R	7.813	0.052	96%
NIL_cure_M1 non-R2R	4.364	0.066	94%
Uncured resins (Reference)			
NIL_cure_M1	7.094	1.72	
XPA	10.221	1.818	

Figure S2. (**A**) Optimization of the UV photocuring power for R2R UV-NIL imprint prepared with NIL_cure_M1 resin (N = 3 for each curing dose). With the exception of the SP probe, we observed a consistent trend of rising chemiluminescence signals as the curing doses increased. Therefore, UV-NIL imprints were prepared with 100% curing power (14 W/cm^2) in the subsequent investigations. (**B**) Determination of double bond conversion (DBC) rates for UV imprints prepared at different UV photocuring doses. A higher percentage of conversion is observed at a higher UV power exposure, affecting the stability of the imprints. (**C**) Stability of UV imprints prepared between high (fully cured) and moderate (semi-cured) photocuring doses. While the water contact angles of a fully cured imprint remained relatively stable over a period of 80 days in ambient storage, the surface energies of a semi-cured imprint fluctuated, resulting in the decrease in the water contact angle by more than 15°.

S3. Revised method for R2R UV-NIL lamination

We introduced a new method of UV lamination to homogenize the surface energies of UV-NIL chips. Previously, UV-NIL chips were bonded with a cover foil pre-coated with a thin layer of liquid (uncured) resin before UV lamination took place. Although this process offered a strong bonding of the peeling force of 21.8 N/mm (Fig. S3 A), it was still achieved with thiol-rich XPA resin, which has different wettability from NIL_cure_M1 used for imprinting, giving rise to the inhomogeneity of surface energies of microfluidic channels. In addition, using liquid resin required its layer to be adequately thin to prevent excess resin from seeping into the channels and impeding the flow.

Thus, we revised the R2R lamination process by switching the laminating resin from XPA to NIL_cure_M1 to homogenize all boundaries of microfluidic structures with one resin. To overcome the effect of O_2 inhibition of NIL_cure_M1 that causes the resin to 'dewet' from the substrate foil within channels regions after bonding, the resin-coated cover foil was partially cured on a flat Ni master at 4 m/min 45% power (6.3 W/cm^2) prior to nitrogen-assisted UV lamination (14 W/cm^2 at 1 m/min) performed under a higher counter pressure of 2 bars to promote better adhesion. Our new approach to R2R lamination with the NIL_cure_M1 resin offered a peeling force of 19.4 N/mm (Fig. S3 A), which is lower than the value formerly achieved with XPA but still sufficient for maintaining leak-free capillary flow. Despite having a higher water-contact angle than XPA, NIL_cure_M1 resin increased the initial flow rate by larger than twofold to 1.10 ± 0.10 µL/s possibly due to a lesser degree of channel obstruction, bringing the flow characteristic closer to that of PSA-bonded chips (Fig. S3 B).

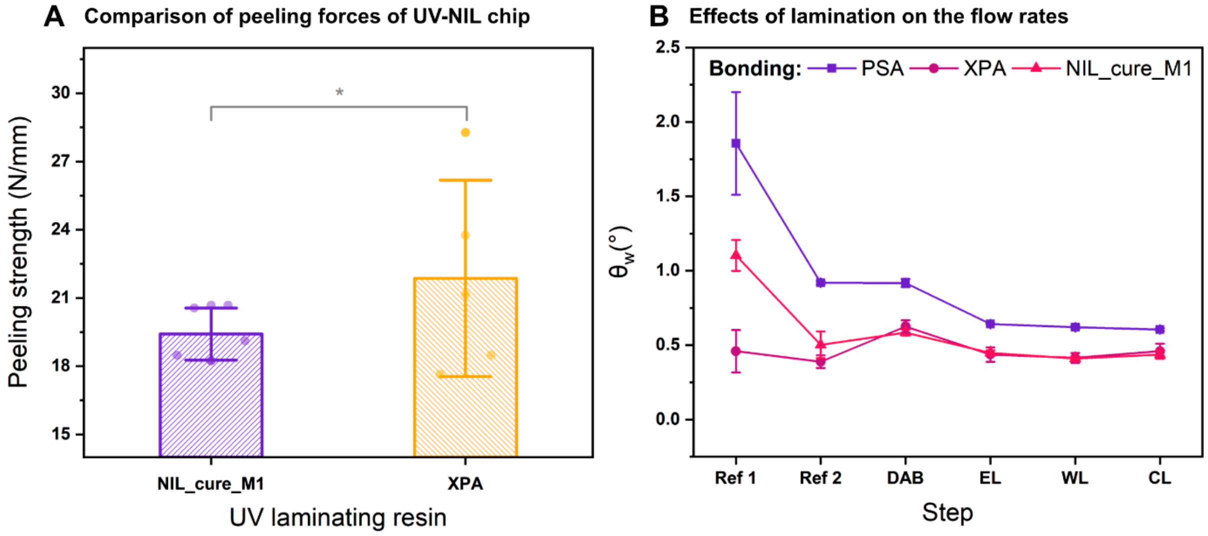

Figure S3. (**A**) Peeling strength of UV-NIL chips prepared with two photocurable resins, NIL_cure_M1 (new approach) and XPA (former approach), which exhibit a higher peeling strength. However, due to the surface energy mismatch of XPA with the imprinting resin (NIL_cure_M1), we re-optimized the bonding process to permit stable chip lamination while still complying to the established R2R setup and retaining a desired flow characteristic. (**B**) Volumetric flow rate of UV-NIL chips achieved with NIL_cure_M1 as a laminating agent (▲) in comparison to the previous variants laminated with PSA (■) and the XPA resin (●).

S4. Evaluation of chemiluminescence signals on 100 meters of UV-NIL foil

After demonstrating the alternative R2R lamination with NIL_cure_M1, the final aspect of the fabrication process that we would like to address is the UV imprinting and lamination of UV-NIL chip production. Previously, the effect of R2R imprinting scale on the implemented µ-ELISA assay had not been investigated. In this setup, we imprinted a total of 100 m of imprint to determine the relationship between the imprinting position and the resulting measured signals (UV-NIL-Opt and EC-Opt in Table S1). We observed a consistent trend of improving chemiluminescence in all test probes from the start of the imprinting position (meter 0) to the end (meter 97) (Fig. S4 A). While other probes exhibited moderate signal improvements, RBD notably showed a significant threefold increase in the signals from the middle positions (meters 25 and 75) to the end position, with the measured values above 10^4 a.u. that had never been achieved on UV-NIL chips (Fig. S4 A). This key information pointed to the possibility that the imprint may carry surface residues that vary along the positions as the imprinting progresses, and the presence of such residues has a direct influence on the chemiluminescence outcomes.

To further scrutinize this effect, we first evaluated the water contact angles along the length of imprint to determine any significant variations in the surface free energies. Although the contact angles fluctuated from one position to the other, their changes were negligible and did not correspond to the pattern of signal increase (Fig. S4 B). We used XPS to determine the atomic fractions of each element on the imprint. The main components of the resin, C and O, were the predominant species and consistently observed in all positions (Table S2). Using XPS allowed us to probe into rare elements that were also present on the imprint, of which we found a trace of fluorocarbon that continued to diminish along the imprinting length (Fig. S4 C). The atomic fractions of F on the imprints of less than 0.05% might originate from fluorinated anti-sticking coating that was applied onto the Ni stamp to form a self-assembly monolayer to help release the cured resin from the stamp (demolding). Depending on the photocurable resin used for imprinting, such treatment with anti-sticking coating may not be required. This finding suggested the possibility of the fluorocarbon from the coating inadvertently contaminating the imprint, affecting the adhesion of probe molecules during the process of passive adsorption. Based on this evidence, it is important to be critically mindful of unnecessary chemical treatments of the Ni stamp before imprinting to avoid leaving undesired surface residues that adversely impact the assay.

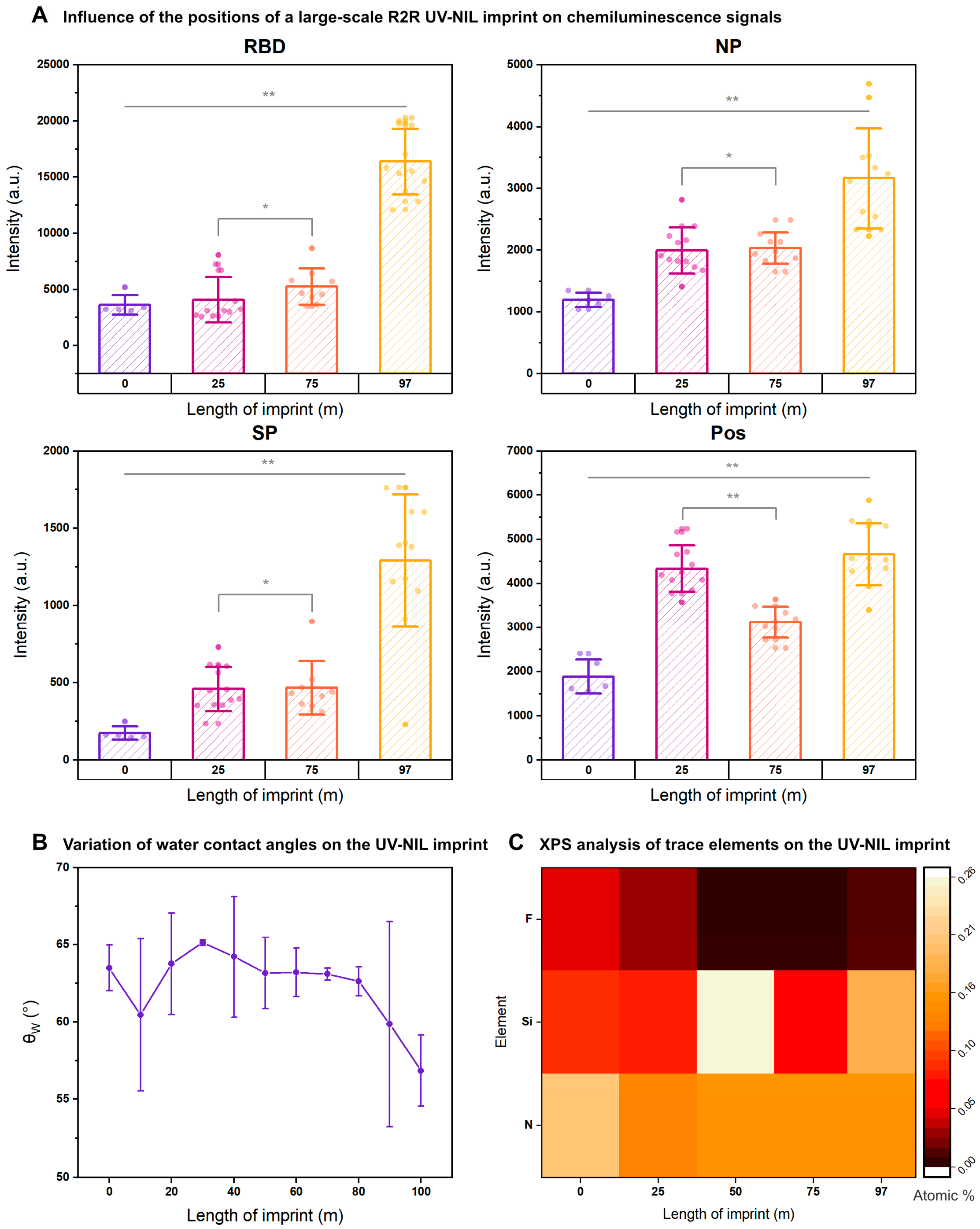

Figure S4. (**A**) Evaluation of the effect of large-scale R2R UV imprinting and the change in UV bonding resin from XPA to NIL_cure_M1 on chemiluminescence outcomes (N = 12 for each position). All COVID-19 test probes exhibited significant signal improvements from the starting (meter 0) to the end (meter 97) positions with comparable signals between meters 25 and 75. (**B**) Water contact angles of the imprint surface at different imprinting length indices exhibited no significant variations at different positions. (**C**) Atomic percentages of trace elements on the imprint surface at different imprinting length indices. The decreasing fraction of fluorine suggests the depletion of fluorinated species over the course of imprinting that spanned 100 meters in total. The potential source of F is anti-adhesion coating that was applied on the Ni master prior to imprinting to assist with demolding. Given the universal increases of chemiluminescence towards high position indices, the presence of F, albeit scarce, *resonated* with the reduction of probe's activ*iti*es. (* denotes $p > 0.05$, ** denotes $p < 0.05$).

S5. Stability of UV-NIL chips in storage

Stability in storage is one of the key merits that dictate the successful development of a diagnostic test kit. As a preliminary investigation, we evaluated the chemiluminescence signals of UV-NIL chips at days 5 and 36 post-biofunctionalization. We stored the chips dedicated for each time point in vacuum pouches with desiccants. The chips were kept refrigerated at 4°C until the time of measurement (UV-NIL-Opt and EC-Opt in Table S1), which demonstrated comparable signals between both time points (Fig. S5).

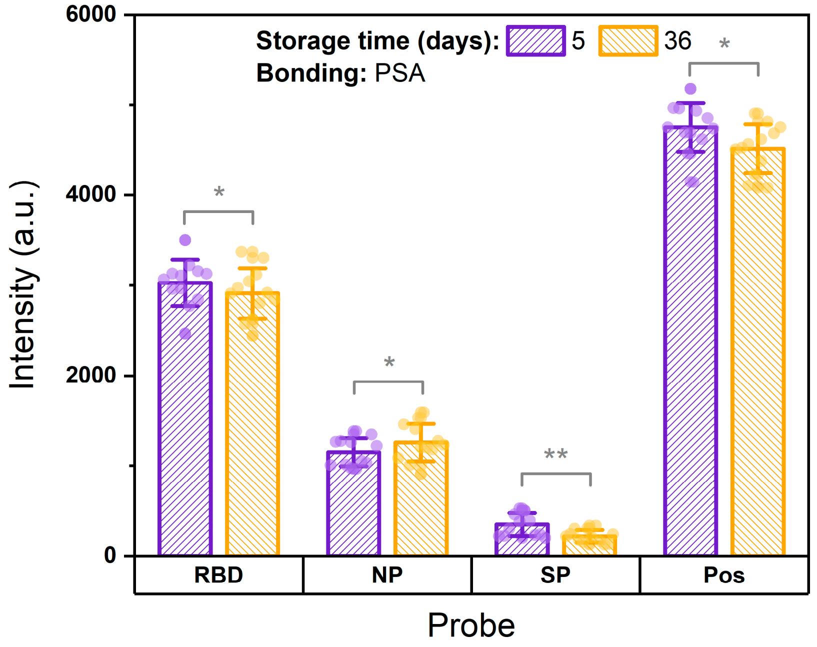

Figure S5. Stability of PSA-laminated UV-NIL chips in 4 weeks of storage at 4°C showing consistent signal developments on all probes (N = 12 for each time point). The imprint was selected from the middle position (meter 50-51) of the 100-m R2R imprint (see evaluation of chemiluminescence signals on 100 meters of UV-NIL foil). (* denotes $p > 0.05$, ** denotes $p < 0.05$).

S6. Time optimization for COVID-19 IgG measurement on the foil chips

We have demonstrated a promising prospect of using foil chips as a platform for implementing a multiplex point-of-care assay in the comprehensive cross-comparison evaluation. Nevertheless, to further advance their maturity and technological readiness, we additionally addressed the value of rapid assay turnaround time. In the previous evaluation, the total time consumption of on-chip measurement steps was nearly 35 min (UV-NIL-Long and EC-Long in Table S1), which equates to 13 chips/device on an 8-hour testing routine. To improve upon the turnaround time by reducing the overall time consumption of the on-chip measurement, we evaluated the signal outcomes of assays that were performed with a shortened protocol (UV-NIL-Opt and EC-Opt in Table S1) that trimmed off more than 10 min from the measurement time, bringing the analysis time closer to the same level as that of the standard IM chips. This optimized protocol yielded lower values of chemiluminescence signals obtained with a longer incubation protocol, yet the effect was largely insignificant. Based on the optimized protocol, both types of the foil chips generated comparable signals (Fig. S6). When implemented, it extends the number of tests from 13 to 20 chips/device over a period of 8 hours of uninterrupted measurements, improving the throughput of the testing capability.

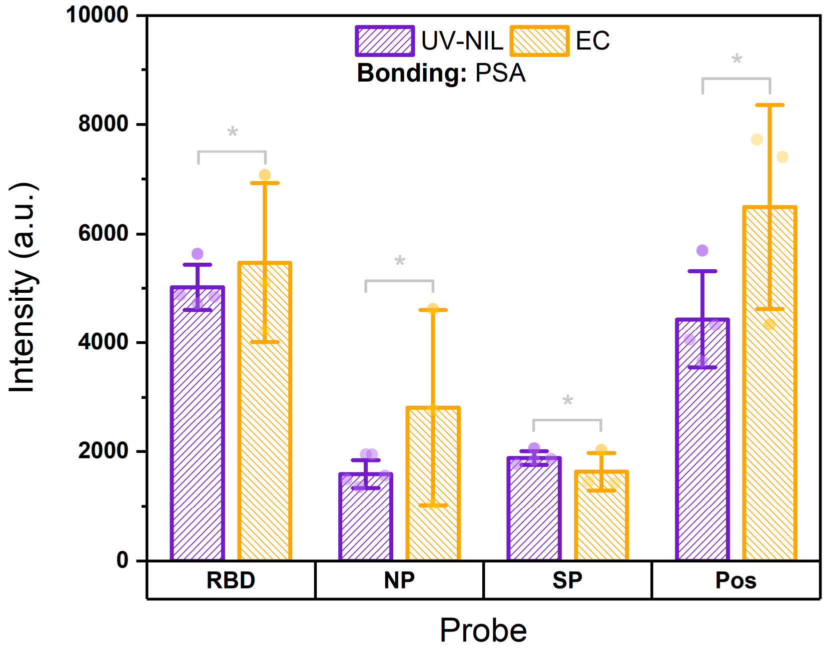

Figure S6. Time optimization of the adjusted measurement protocol for UV-NIL (N = 4) and EC (N = 3) chips laminated with pressure-sensitive adhesive (PSA). Time-optimized measurement could be completed with a shorter total incubation, which brings the on-chip analysis time to under 30 minutes. (* denotes p > 0.05, ** denotes p < 0.05).

S7. Visualization of liquid flow in microfluidic chip geometry

The image sequence in Fig. S7 visualizes liquid flow within the microfluidic chip geometry using a water-based dyed solution (methylene blue). A drop of liquid, applied directly at the inlet, is automatically drawn into the reaction channel via capillary action. Once the applied liquid at the inlet is completely consumed, the flow continues with the next dispensing step, typically performed by the automated Genspeed analyzer. After the reaction channel is fully filled, suction continues towards the capillary pump reservoirs located on both sides of the chip until the reservoirs are fully occupied with liquid.

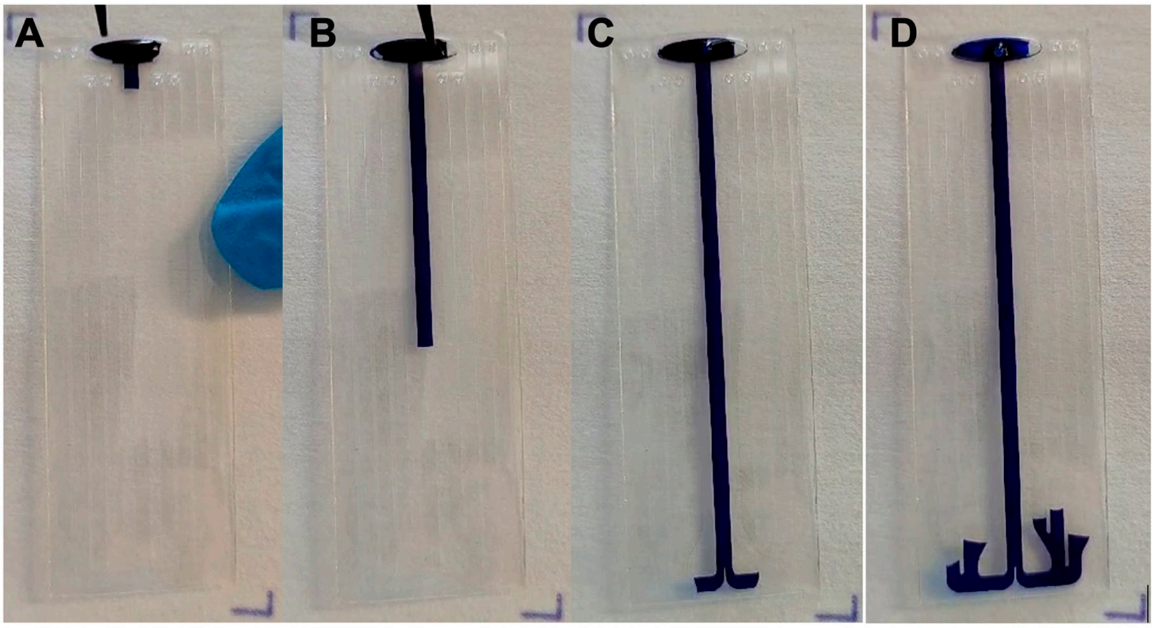

Figure S7. Liquid flow in the microfluidic chip geometry. (**A**) After the first pipetting, the liquid in the chip inlet begins to be drawn into the reaction channel by capillary action. (**B**) Once the inlet is emptied, a second dispensing is required for the continuation of liquid flow. (**C**) and (**D**) The sample channel is completely filled, and the capillary pump reservoirs are in the process of filling.

S8. Adjusted measurement protocols for COVID-19 IgG detection of foil chips

Both variants of the foil chips have significantly lower fluid capacities than the standard IM chip from GENSPEED. Therefore, we adjusted the measurement protocol to enable on-chip chemiluminescence detection of the target IgG samples (reference solutions). For each foil chip variant, the measurement was performed with the reagent volumes specified in the highlighted columns. To compensate for smaller volumes used, longer incubation time (Long) was introduced to improve the signal development. However, we later demonstrated that a shorter incubation time (Opt) could also be used to achieve comparable signals under less total assay time.

Table S1. Measurement protocols for foil chips.

Reagent	Standard (Total volume = 147 µL)		UV-NIL (Total volume = 127 µL)				EC (Total volume = 89 µL)			
	Volume (µL)	Time (m:s)	Volume (µL)	Time (m:s)			Volume (µL)	Time (m:s)		
				Short	Long	Opt		Short	Long	Opt
Reference 1	12.5	2:30	12.5	2:30	5:00	4:00	6.5	2:30	5:00	4:00
Reference 2	12.5	2:30	12.5	2:30	5:00	4:00	6.5	2:30	5:00	4:00
Solution B	25	5	25	5:00	10:00	7:00	12.5	5:00	10:00	10:00
EL	27	5	27	5:00	10:00	7:00	13.5	5:00	10:00	10:00
WL	10×4	1:00×4	5×4	1:00×4	1:00×4	1:00×4	5×4	1:00×4	1:00×4	1:00×4
CL	30	-	30	-	-	-	30	-	-	-

S9. XPS analysis of R2R UV-NIL imprint

Trace elements on the imprint surface were determined by X-ray photoelectron spectroscopy (XPS) by performing survey scans (high intensity–low resolution) of the whole spectral range in combination with a highly prolonged integration time on specific positions. The detailed scans of the main element peaks with higher resolution but lower intensity were used for the peak fitting and data processing.

Table S2. Evaluation of residual elements at different position indices of a large-scale R2R UV imprint by X-ray photoelectron spectroscopy (XPS) analysis

Element	Meter 0	Meter 25	Meter 50	Meter 75	Meter 100
C	77.33	77.5	77.06	77.51	77.7
O	22.34	22.25	22.54	22.27	21.95
N	0.2	0.13	0.15	0.15	0.15
Si	0.09	0.08	0.26	0.06	0.18
F	0.05	0.03	Undetectable	Undetectable	0.01
